# Differences in Sleep EEG Coherence and Spindle Metrics in Toddlers With and Without Language Delay: A Prospective Observational Study

**DOI:** 10.21203/rs.3.rs-3904113/v1

**Published:** 2024-02-14

**Authors:** Xinyi Hong, Cristan Farmer, Nataliia Kozhemiako, Gregory L Holmes, Lauren Thompson, Stacy Manwaring, Audrey Thurm, Ashura Buckley

**Affiliations:** National Institute of Mental Health Division of Intramural Research Programs: National Institute of Mental Health Intramural Research Program; National Institute of Mental Health Intramural Research Program; Brigham and Women’s Hospital; University of Vermont Department of Medicine; Washington State University Elson S Floyd College of Medicine; University of Utah Department of Communication Sciences and Disorders; National Institute of Mental Health Intramural Research Program; National Institute of Mental Health Intramural Research Program

**Keywords:** Sleep architecture, brain development, cognitive function, neurophysiology, diagnostic markers, language delay

## Abstract

**Background:**

Sleep plays a crucial role in early language development, and sleep disturbances are common in children with neurodevelopmental disorders. Examining sleep microarchitecture in toddlers with and without language delays can offer key insights into neurophysiological abnormalities associated with atypical neurodevelopmental trajectories and potentially aid in early detection and intervention.

**Methods:**

Here, we investigated electroencephalogram (EEG) coherence and sleep spindles in 16 toddlers with language delay (LD) compared with a group of 39 typically developing (TD) toddlers. The sample was majority male (n = 34, 62%). Participants were aged 12-to-22 months at baseline, and 34 (LD, n=11; TD, n=23) participants were evaluated again at 36 months of age.

**Results:**

LD toddlers demonstrated increased EEG coherence compared to TD toddlers, with differences most prominent during slow-wave sleep. Within the LD group, lower expressive language skills were associated with higher coherence in REM sleep. Within the TD group, lower expressive language skills were associated with higher coherence in slow-wave sleep. Sleep spindle density, duration, and frequency changed between baseline and follow-up for both groups, with the LD group demonstrating a smaller magnitude of change than the TD group. The direction of change was frequency-dependent for both groups.

**Conclusions:**

These findings indicate that atypical sleep EEG connectivity and sleep spindle development can be detected in toddlers between 12 and 36 months and offers insights into neurophysiological mechanisms underlying the etiology of neurodevelopmental disorders.

**Trial registration:**

https://clinicaltrials.gov/study/NCT01339767; Registration date: 4/20/2011

## Background

Sleep is crucial for the development of physiological and cognitive processes in early childhood. It plays a key role in early language development, with studies examining word learning reporting sleep to be crucial in facilitating consolidation, retention, and recall^1,2^. Sleep disturbances are common in infants and toddlers with neurodevelopmental disorders and have been found to be associated with impairments in language development in populations known to be at risk for language and other delays, such as Down’s syndrome, Williams syndrome, and Fragile X syndrome^3–5^.

Although there are many neuroimaging studies investigating the functional and structural underpinnings of neurodevelopmental disorders, few studies have capitalized on the potential of sleep encephalogram (EEG) to examine the developing brain. Sleep electroencephalogram (EEG) is highly advantageous for the study of neurodevelopmental disorders in young children due to its temporal resolution, non-invasiveness, and reproducibility. Furthermore, sleep EEG (as opposed to resting-state EEG) allows for neural activity to be captured in a way that is unaffected by signal noise from external waking stimuli. This allows for a more sensitive and robust way to image early circuit development, particularly in very young children for whom it may otherwise be difficult to do so. As such, examining sleep micro-architecture can offer key insights into neurophysiological abnormalities that are associated with atypical neurodevelopmental trajectories and can aid in early detection and intervention. Since language delays are one of the earliest behavioral signs of neurodevelopmental delays brought to the attention of providers^6^, this study aims to elucidate the sleep-unique neural underpinnings of neurodevelopmental disorders by examining sleep EEG coherence and sleep spindles in toddlers with and without language delay.

### EEG Coherence

Functional brain connectivity is the degree of temporal correlation between two spatially distant neuronal populations; as such, it possibly allows for conclusions to be made regarding functional interactions between two or more brain regions^7^. Functional connectivity can be ascertained via EEG imaging by examining the statistical relationship between two or more electrodes on a frequency-by-frequency basis. The relationship between the phase shift and amplitude ratio of the two signals is analyzed over time, with higher consistency between the signals being regarded as strong coherence, or proof of functional connectivity^8^.

EEG coherence has been examined in several studies of the neuronal underpinnings of language systems development^9^. Because language development requires the coordination of complex neural networks, EEG coherence is a relevant tool in examining the functional connectivity of the brain as it is undergoing crucial maturational periods. Frequency-by-frequency analysis has resulted in speculation about frequency specificity in the development of language neural networks. For instance, slow-wave activity in the delta frequency has been found to be associated with grouping words into syntactical phrases, the beta band has been suggested to play a role in top-down predictive functions in language processing, and oscillations in the alpha range may play a role in verbal working memory^10^.

Altered functional connectivity has been found to be a common attribute in children with neurodevelopmental impairments^11^. Most published work investigating aberrations in functional connectivity associated with neurodevelopmental disorders focuses on autism spectrum disorder (ASD). Evidence for the directionality of differences in functional connectivity have been mixed: studies report both lower^12–14^ and increased^15–17^ coherences in individuals with ASD relative to non-ASD controls. The heterogeneity of study results is likely attributed to the various states and regions examined across studies, as well as differences in the composition of comparison groups. Studies in ASD have also found altered functional connectivity in language networks. Among a sample of 9-month-olds, hypoconnectivity within language and communication-related networks predicted impaired communication skills later in childhood^18^. Another longitudinal study found that whilst infants at low risk for ASD demonstrated increasing connectivity between frontotemporal language regions during the first year of life, infants at high familial risk for ASD demonstrated more static profiles in their functional connectivity development^19^. Furthermore, Linke et al.^20^ found in children with ASD increased connectivity between the thalamus and Heschl’s gyrus, an area which has been implicated in auditory processing and language learning^21,22^.

While most of the aforementioned studies utilize resting-state fMRI or resting-state EEG to conduct coherence analyses, only a few have specifically explored *sleep* EEG connectivity. In one instance, researchers found decreased during NREM sleep intra-hemispheric coherence in frontal areas and decreased coherence within the right hemisphere in children and adolescents with Asperger syndrome^23^. These findings were most prominent for delta, theta, alpha, and sigma frequency bands. Similarly, EEG coherence during REM sleep in adults with autism showed increased intra-hemispheric communication among left visual cortex and other regions near or distant from the occipital cortex, while reduced coherence involved right hemisphere frontal electrodes^24^. Another study in children with autism spectrum disorders found lower synchronization in the EEG brain dynamics during NREM sleep, which in turn supports the underconnectivity model in autism^25^. Finally, a study in 2015 comparing brain connectivity in children aged 2 to 6 years with autism, developmental delay without autism, and typically developing controls found that increased coherence during slow-wave sleep distinguished children with autism from both neurotypical and developmentally delayed children, suggesting distinct differences in sleep EEG functional connectivity unique to autism^26^.

### Sleep Spindles

Sleep spindles are a hallmark of NREM sleep presenting as brief bursts of waxing-and-waning oscillations between 10 to 16 Hz^27^. Sleep spindles are believed to have functional roles in information processing, memory consolidation, and neuroplasticity during development^28–30^. Because spindle properties are known to change throughout the lifespan, they can provide insight into the neuro-maturational trajectories of individuals with neurodevelopmental disorders^31^. However, despite the high prevalence of sleep problems in infants and toddlers with neurodevelopmental disorders, few studies have examined sleep spindle properties in this population. There is preliminary evidence that sleep spindle properties are different in children with developmental impairments. For example, Shibagaki et al. found that children aged 3 months to 8 years with intellectual disability who exhibited lower spindle density had lower developmental quotient scores^32^. Tessier et al. found shorter spindle duration and decreased spindle density in children with autism aged 6–13 years compared to typically developing children aged 7–12 years^33^. In 2018, Farmer et al. found reduced spindle density in children with ASD aged 2–6 years compared to age-matched typically developing controls and children with developmental delay without ASD^34^. Similarly, a recent paper by Mylonas et al. reported both decreased spindle density in ASD relative to controls and a disruption in slow oscillation spindle coordination during N2 sleep in participants aged 9–17 years^35^. However, due to the paucity of longitudinal data in early childhood, differences in developmental trajectories of sleep spindles in children with and without neurodevelopmental disorders are currently unknown. Furthermore, the authors did not identify any studies which have investigated spindle frequency and chirp.

Additionally, while previous research has primarily focused on sleep spindle density in neurodevelopmental disorders, limited studies have explored other spindle metrics, including spindle frequency and chirp (intra-spindle frequency change). Investigating spindle frequency and chirp can potentially add valuable insight, as these oscillations reflect thalamo-cortical activity, and their internal frequency variation can hold relevant information for understanding neurophysiology and exploring brain pathology in neurodevelopmental disorders^36^. By examining spindle density, frequency, and chirp in combination, this study aims to provide a more comprehensive assessment of the sleep spindle features in early childhood, facilitating the identification of potential markers for language and cognitive impairments, and contributing to our understanding of the neural substrates underlying these conditions.

Given these gaps, here we present an exploratory analysis that evaluates EEG coherence during wake, rapid-eye movement (REM), and slow-wave sleep (SWS) stages, and sleep spindle density, duration, frequency, and chirp at two time points in a cohort of toddlers with language delay in comparison to typically developing toddlers. Children with language delay were the target population of this study due to their risk of a later ASD diagnosis. Thus, in addition to between-group differences, we explored within-group relationships of these EEG features to dimensional behavioral markers of neurodevelopment. We hypothesized that toddlers with language delay would exhibit higher EEG coherence compared to typically developing toddlers, and that these differences would be state-dependent. Additionally, we predicted that toddlers with language delay would demonstrate decreased density, frequency, and chirp compared to typically-developing toddlers, and that they will demonstrate a smaller magnitude of change in these sleep spindle properties between baseline and follow-up.

## Methods

### Participants

Participants were drawn from a pilot natural history protocol conducted at the NIH Clinical Center in Bethesda, Maryland, USA (protocol number 11-M-0144; NCT01339767). The study was approved by the NIH Institutional Review Board. Consent from the parent or guardian of each child was obtained prior to participation. Inclusion criteria were being between the age of 9 of 21 months, born full-term, no significant medical or motor impairment deemed responsible for the delays (or any known genetic disorder for the typically developing group), and English reported as the primary language spoken in the home. Additionally, at the initial evaluation, eligibility for the language delay (LD) group included receptive and expressive language scores in the very low range (T scores ≤ 30) on the Mullen Scales of Early Learning^37^ (MSEL), and eligibility for the typically developing (TD) group included scores within1.5 standard deviations (SDs) of the mean on all domains of the MSEL.

A total of 62 (LD, n = 19; TD, n = 43) toddlers enrolled in the natural history study. As described below, the analysis sample for the present study that included analyzable sleep studies was n=55 toddlers (LD, n = 16; TD, n = 39) aged 12 to 22 months at their first visit (mean age 17.4±2.8 months) (Table 1). The sample was majority male (n = 34, 62%) and white (n = 36, 65%). Seven (41%) of the LD participants did receive a research ASD diagnosis (based on clinical judgment of DSM-5 criteria informed by research-reliable administrations of the Autism Diagnostic Interview and Autism Diagnostic Observation Schedule) during their study participation. This small sample size precluded investigation of relationships to an ASD diagnosis.

### Procedures

The study design was longitudinal, comprising study visits at 12 and/or 18 months (depending on age of study entry), 24 months, and 36 months. At each study visit, participants received a neurodevelopmental assessment supervised by doctoral level clinicians, including the MSEL and Vineland Adaptive Behavior Scales, 2^nd^ Edition^38^. Two scores were computed from the MSEL and used in the current analysis:, the Verbal Developmental Quotient (VDQ; using the Receptive and Expressive subscales) and the Nonverbal Developmental Quotient (NVDQ; using the Fine Motor and Visual Reception subscales). DQs are analogs of IQs and are calculated by averaging the age equivalents from each subscale, dividing by the chronological age, and multiplying the result by 100. From the Vineland-II, the Socialization standard score was used to reflect a continuous metric of social skills relative to normative expectations. The Socialization score is norm-referenced with a population mean of 100 and SD of 15.

**Figure 1:**
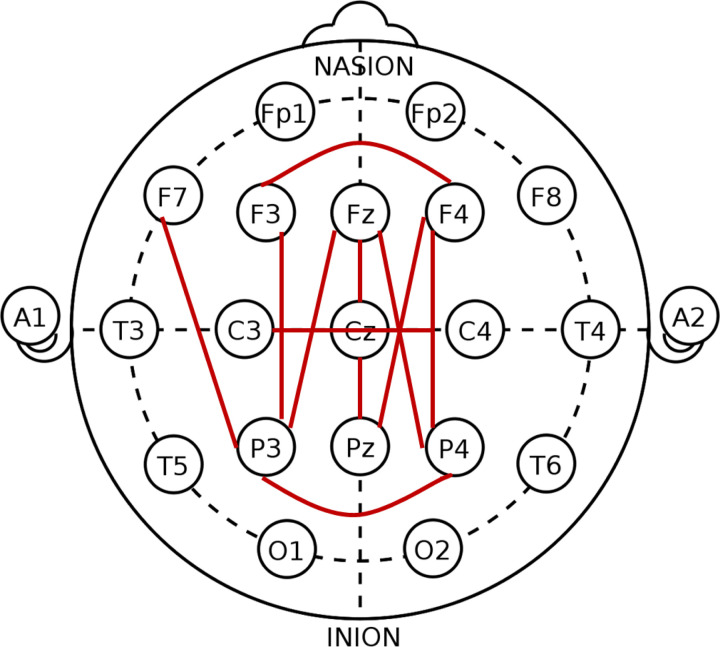
Electrode placement. Red lines indicate electrode pairs pulled for coherence analysis.

An optional overnight sleep EEG was performed at the participants’ first visit (baseline; BL) and at the 36-month visit (follow-up; FU); those with valid sleep EEG data were included in the current analysis. A total of 34 (LD, n=11; TD, n=23) participants had both BL and FU sleep studies, while 21 (LD, n=5; TD, n=16) had only BL. Digital EEGs were recorded during awake, drowsy, and sleep states using the 10–20 system of electrode placement (Fig. 1).

### Analysis

#### EEG Coherence.

“Clean” ten-minute segments of awake, slow-wave sleep (SWS), and rapid eye movement (REM) sleep were selected for coherence analysis, which was performed masked to participant group using Neuroguide software (Applied Neuroscience Inc., St. Petersburg, FL) using a linked-ear montage.

Select electrode pairs were pulled out for coherence analysis based on findings from Buckley et al.^26^ which found increased coherence in children with ASD compared to typically developing controls, concentrated in frontal-parietal pairs. Electrode pairs selected for analysis are F3-P3, F4-P4, F4-Pz, P3-Fz, P4-Fz, P3-F7, C3-C4, Cz-Pz, F3-F4, Fz-Cz, and P3-P4 (Fig. 1). Frequency bands selected for coherence analysis are Delta (1–4 Hz), Theta (4–8 Hz), Alpha 1 (8–10 Hz), Alpha 2 (10–12 Hz), Beta 1 (12–15 Hz), Beta 2 (15–18 Hz), and Beta 3 (18–25 Hz).

Coherence values were subjected to Fisher transformation and fixed effects of group (LD versus TD) and age in months (centered at 18 months) were evaluated with the lme4^39^ package for R version 4.2.1^40^ using a linear mixed model with a random subject-level intercept. In separate exploratory analyses, we evaluated the relationship between coherence and behavioral outcomes (MSEL VDQ and NVDQ, and Vineland-II Socialization standard score) by adding the mean-centered behavioral score as a fixed effect. Because these variables are aliased with group membership, we included an interaction between group and behavioral variable, and evaluated the slope of the behavioral variable on coherence within each group to avoid confounding.

#### Spindle Properties.

Both slow spindles (9 Hz and 11 Hz) and fast spindles (13 Hz and 15 Hz) were evaluated separately for density (number of spindles per minute), amplitude, duration, frequency (Hz), and chirp (intra-spindle frequency change). Spindle data were analyzed from all 16 channels using Luna^41^, a software package developed at Harvard Medical School designed to analyze polysomnogram (PSG) recordings and automate spindle analysis. The Luna software utilized a Morlet wavelet transformation method of spindle detection. Spindle frequency, amplitude, duration and epoch counts were recorded for each spindle and plotted on a graph for visual analysis and verification.

The effect of age on spindle features was evaluated using linear mixed effects models with fixed effects for study visit (BL vs. FU), group, and sex, and a random subject-level intercept. The relationship of spindle density to behavioral scores were evaluated as described above for coherence.

Given the volume of statistical tests performed in this analysis, we used a graphical approach to summarizing results and a p-value threshold of .01 to determine which effects to focus upon for discussion. However, because these were exploratory analyses, we did not adjust p-values for multiplicity. The complete set of uncorrected p-values and test statistics is available as supplementary materials.

## Results

### Coherence

**Figure 2:**
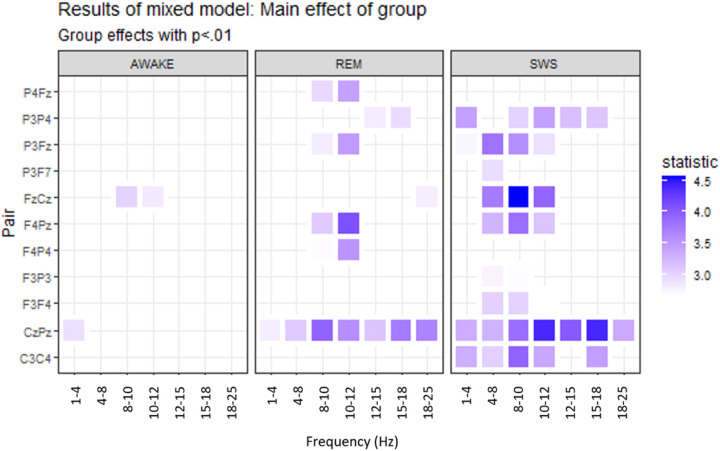
Differences in coherence between LD and TD. Dark blue indicates higher coherence for LD. P3-F7 did not have differences in coherence that reached p<.01 for any of the frequencies selected and is thus not displayed.

Group differences in coherence during awake, REM, and SWS states are shown in Fig. 2. Increased coherence was observed for LD relative to TD, with differences being most prominent during SWS. Few differences in coherence between LD and TD were observed during the awake state.

Results for the effect of age on coherence were evaluated. Increased coherence with age was found for four electrode pairs (C3-C4, F4-Pz, Fz-Cz, and P3-Fz), all within the beta 1 frequency (12–15 Hz). Decreased coherence with age was only found for one frequency/pair combination, F3-F4 in the delta frequency. The group-by-age interactions indicated that the effects of age on coherence did not appear to differ between groups.

**Figure 3:**
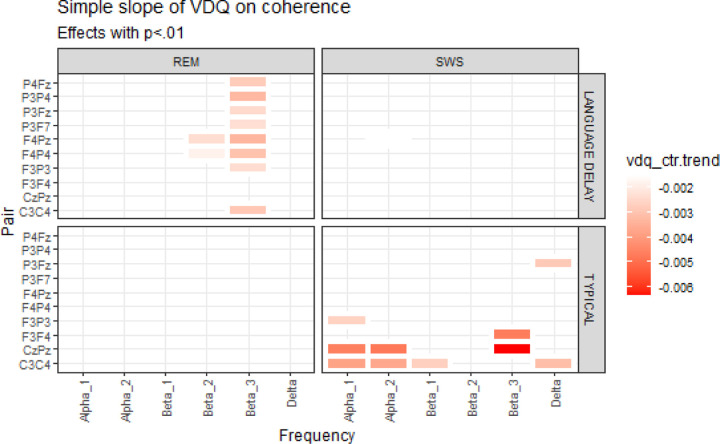
Simple slope of VDQ on coherence. Red boxes indicate a negative relationship between VDQ and coherence for the pairs and frequencies shown.

Within-group relationships between coherence and behavioral scores (NVDQ, VDQ, and Socialization) were evaluated in separate models. No relationships between scores and coherence were found for NVDQ or socialization. Within the LD group, lower VDQ scores were related to higher REM coherence in the beta 3 frequency. Similarly, within the TD group, lower VDQ scores were related to higher SWS coherence (Fig. 3).

### Spindle Properties

#### Density.

**Figure 4:**
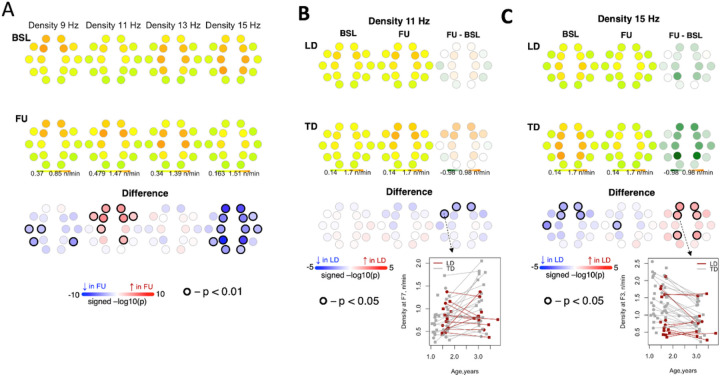
Density of spindles between baseline and follow-up visits. Panel A shows density of spindles between baseline and follow-up for the entire sample. Panel B shows differences in changes in density from baseline to follow-up between LD and TD for 11 Hz spindles. Panel C shows differenes in changes in density from baseline to follow-up between LD and TD for 15 Hz spindles.

In the combined sample, density of slow spindles at 9 Hz decreased from BL to FU in central and temporal channels. Density of slow spindles at 11 Hz increased from BL to FU in frontal channels (Fig. 4). This increase was greater for TD compared to LD. Furthermore, density of fast spindles at 15 Hz decreased from BL to FU across multiple channels (Fig. 4). At 15 Hz, spindle density was lower for LD than TD in frontal channels at BL; this difference was smaller in magnitude at FU (Fig. 4). As an exploratory analysis, we evaluated the relationship of NVDQ, VDQ, and Socialization to density within each study group. The parameter estimates tended to be negative, associating higher density with lower behavioral scores, but the confidence intervals were wide and contained zero. The single exception was for the relationship of Socialization in the LD group to spindle density in 15 Hz at the T6 electrode.

#### Duration.

**Figure 5:**
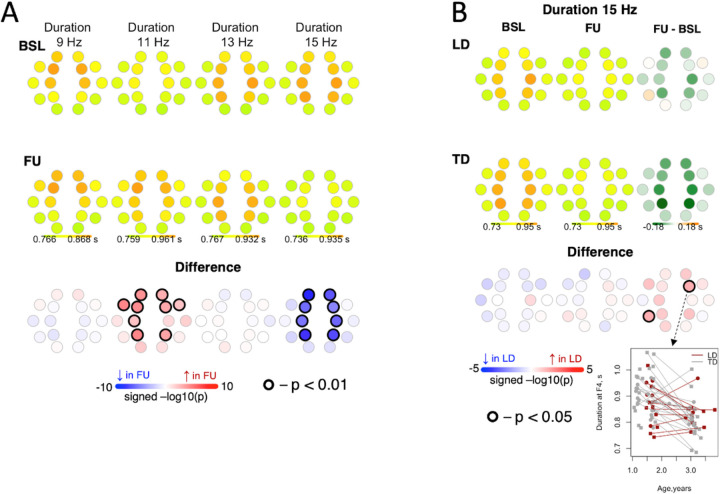
Differences in duration of spindles between baseline and follow-up. Panel A shows differences in duration of spindles between baseline and follow-up for the entire sample. Panel B shows differences in changes in duration from baseline to follow-up between LD and TD for 15 Hz spindles.

In the combined sample, duration of slow spindles at 11 Hz increased from BL to FU across multiple channels, whereas duration of fast spindles at 15 Hz decreased from BL to FU across multiple channels (Fig. 5). This decrease was lower in magnitude for LD compared to TD.

#### Frequency.

**Figure 6:**
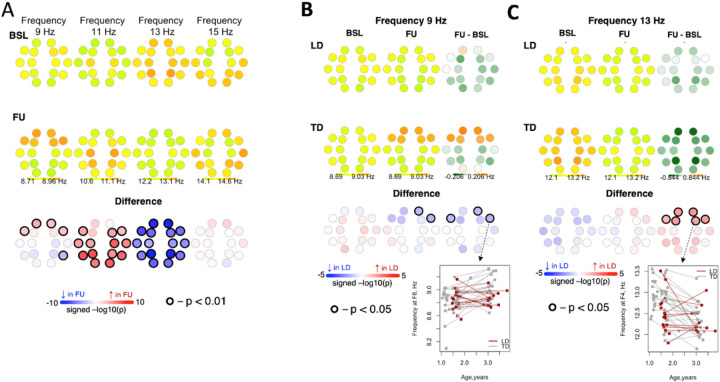
Differences in frequency of spindles between baseline and follow-up. Panel A shows differences in frequency of spindles between baseline and follow-up for the entire sample. Panel B shows differences in frequency from baseline to follow-up between LD and TD for 9 Hz spindles. Panel C shows differences in frequency from baseline to follow-up between LD and TD for 13 Hz spindles.

In the combined sample, frequency of slow spindles at 11 Hz increased from BL to FU in posterior channels. Frequency of slow spindles at 9 Hz increased from BL to FU in frontal channels, and this increase was lower in magnitude for LD compared to TD (Fig. 6) Frequency of fast spindles at 13 Hz decreased from BL to FU across all channels.. This decrease was lower in magnitude for LD compared to TD.

#### Chirp.

**Figure 7:**
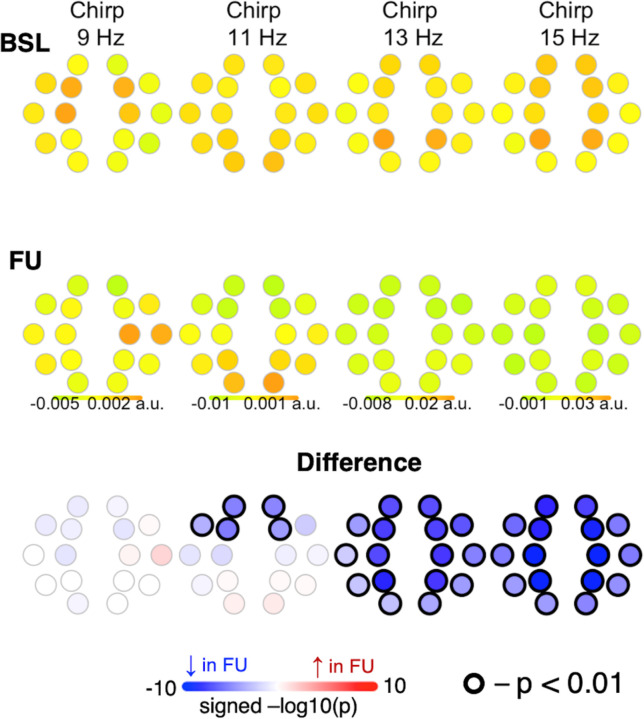
Differences in chirp (intra-spindle frequency change) from baseline to follow-up.

Chirp of slow spindles at 11 Hz and fast spindles at 13 and 15 Hz decreased from BL to FU, with no difference between TD and LD (Fig. 7).

## Discussion

We investigated EEG coherence and sleep spindle properties at two time points in toddlers with and without language delay. Increased coherence found for toddlers with LD was state-dependent, with differences being most prominent during SWS in central electrode pairs across frequency bands. Few changes in coherence were observed over time, indicating that any differences between LD and TD may have already been present prior to toddler age. Within-group analyses indicated that lower VDQ scores were associated with higher coherences within REM only for toddlers in the LD group and within SWS only for children in the TD group. Furthermore, changes in spindle properties were observed between baseline and follow-up for both groups; however, the LD group demonstrated a smaller magnitude of change in spindle density, duration, and frequency in comparison to the TD group. Taken together, these findings support previous findings by Liu et al.^19^ that suggest young children at risk for neurodevelopmental disorders may demonstrate more static neurodevelopmental profiles. In this case, the children were not only at risk for later neurodevelopmental disorders due to their language delay, but they already had delays that necessitated referral.

Interestingly, most of the differences in coherence between LD and TD were detected in the alpha frequency bands. A previous study by Orekhova et al.^17^ found alpha range hyper-connectivity in 14-month-old high-risk infants who were later diagnosed with ASD at 3 years old, suggesting that alpha hyper-connectivity may be an important attribute of ASD neurophysiology. Previous research has indicated that alpha oscillations detected by scalp EEG may contain signals from thalamocortical interactions^43^, which has been speculated by Linke et al.^20^ to play a crucial role in sleep disruptions in ASD. Alpha oscillations also appear to play an important role in language development and have been found to be correlated with typically developing children’s oral language abilities^44^. In this sample, differences in alpha-frequency coherence between LD and TD may indicate abnormal thalamocortical functioning in LD children with increased likelihood for ASD and may also play a role in differences in language skill and trajectory of attainment.

This was an exploratory analysis intended to generate hypotheses for future confirmatory study. Results must be interpreted with respect to several limitations. The study from which these data were drawn was a pilot study, and as such, the sample size is small. Further, since the LD sample generally enrolled at later ages than the TD toddlers, there were chronological age differences between the groups (TD 0.22 years younger than LD on average at baseline). Finally, although the longitudinal nature of this study is a significant strength, attrition led to smaller sample sizes at the later time point and only two time points were available.

## Conclusions

In conclusion, our study of toddlers with language delay reveals early differences in sleep unique electrophysiologic signatures, specifically spindle properties and EEG coherences, potentially serve as biomarkers for neurodevelopmental risk. These findings underscore the significance of investigating sleep-related brain activity to understand language development and neurodevelopmental disorders. Sleep EEG presents a promising avenue to elucidate neural connectivity patterns during critical developmental stages, offering unique insights into the functional maturation of brain circuits that influence language processing and cognitive development.

By identifying potential markers for language delay and neurodevelopmental impairments, our study also highlights the importance of early detection and monitoring. The state-dependent increases in EEG coherence, particularly during slow-wave sleep, emphasize the relevance of sleep-related brain activity as a window into neural connectivity underlying language delay. Longitudinal assessments indicate that certain neurophysiological differences may be present as early as 18 months, supporting the value of early intervention for children at risk for neurodevelopmental disorders. Nevertheless, given the pilot nature of this study and small sample size, further research with larger cohorts is necessary to validate and expand on these results. By unraveling the complexities of sleep-related brain activity, we gain insights into the neural substrates shaping language development and neurodevelopmental trajectories, facilitating targeted interventions and support strategies for optimal developmental outcomes.

## Supplementary Material

Supplement 1

## Figures and Tables

**Table 1.** Sample characteristics.

	Typically Developing (TD)		Language Delayed (LD)	
	Baseline	Follow-up	Baseline	Follow-up
Total N	39	23	17	11
Male sex, n(%)	24 (62%)	15 (65%)	11 (65%)	7 (64%)
Age in years, mean (SD)	1.43 (0.25)	3.10 (0.11)	1.65 (0.11)	3.19 (0.28)
Age range in years	1.05 – 1.78	2.91 – 3.34	1.48 – 1.85	2.82 – 3.82
Ethnicity, n(%)				
Latino or Hispanic	4 (10%)	2 (9%)	2 (12%)	2 (18%)
Not Latino or	35 (90%)	21 (91%)	14 (82%)	9 (82%)
Hispanic				
Unknown	0 (0%)	0 (0%)	1 (6%)	0 (0%)
Race, n(%)				
Black or African-American	3 (8%)	2 (9%)	6 (35%)	4 (36%)
Asian	2 (5%)	1 (4%)	1 (6%)	0 (0%)
White	30 (77%)	18 (78%)	9 (53%)	7 (64%)
Multiple races	4 (10%)	2 (9%)	1 (6%)	0 (0%)
Behavioral scores, mean (SD)				
NVDQ	115.70 (13.81)	113.67 (12.86)	88.41 (19.58)	85.25 (16.62)
VDQ	99.84 (10.63)	108.60 (12.86)	46.77 (16.06)	76.64 (23.19)
Vineland-II Socialization	101.81 (6.05)	105.55 (7.19)	86.35 (6.13)	84.38 (9.81)
ASD diagnosis, n(%)		0 (0%)		7 (41%)

NVDQ = Non-Verbal Developmental Quotient. VDQ = Verbal Developmental Quotient

## Abbreviations

EEG: electroencephalogram
TD: typically developing
LD: language delay
REM: rapid-eye movement
ASD: autism spectrum disorder
fMRI: functional magnetic resonance imaging
NREM: non-rapid-eye movement
SWS: slow-wave sleep
NIH: National Institutes of Health
MSEL: Mullen Scales of Early Learning
DSM-5: Diagnostic and Statistical Manual of Mental Disorders, Fifth Edition
VDQ: Verbal Developmental Quotient
NVDQ: Non-Verbal Developmental Quotient
DQ: Developmental Quotient
IQ: Intelligence Quotient
BL: baseline
FU: follow-up
PSG: polysomnogram
