# Supplementary material for: Differences in Sleep EEG Coherence and Spindle Metrics in Toddlers With and Without Language Delay: A Prospective Observational Study
Source: Res Sq. 2024 Feb 14:rs.3.rs-3904113. Preprint. [Version 1] doi: 10.21203/rs.3.rs-3904113/v1 (PMC10896365; doi:10.21203/rs.3.rs-3904113/v1)
Supplement: Supplement 1 [file NIHPPRS3904113v1-supplement-1.pdf]

Supplementary Files

This is a list of supplementary files associated with this preprint. Click to download.

- STable1Descriptions.docx
- STable1.xlsx
- STable2.docx
